# Variations in gut bacterial communities between lesser white‐fronted geese wintering at Caizi and Shengjin lakes in China

**DOI:** 10.1002/mbo3.1037

**Published:** 2020-03-23

**Authors:** Gang Liu, Zhizhong Gong, Qingyue Li

**Affiliations:** ^1^ School of Life Sciences Anhui Medical University Hefei China

**Keywords:** 16S rRNA gene, *Anser erythropus*, gut bacterial community, variation

## Abstract

The avian gut microbiota plays an important role in shaping the health of its host. However, knowledge of gut bacteria in birds lags behind that of other animals. In this study, we investigated the gut bacterial communities of lesser white‐fronted geese (*Anser erythropus*) wintering at Shengjin Lake and Caizi Lake, China, using high‐throughput sequencing (Illumina MiSeq). Altogether, 1,053,624 high‐quality sequences and 4,405 operational taxonomic units (OTUs) were acquired from 30 fecal samples (15 per lake). The OTUs represented eight phyla and 17 classes from the Caizi Lake samples and seven phyla and 16 classes from the Shengjin Lake samples. Firmicutes, Proteobacteria, Actinobacteria, and Bacteroidetes were the dominant phyla. The spatial distance and the Chao1, Simpson, and Shannon indices showed that the alpha diversity differed significantly between the samples from both lakes. The phylogenetic tree and heatmap analyses showed that all the Caizi Lake samples were clustered together and all the Shengjin Lake samples were clustered together. These findings suggest that diet may be an important driver of gut microbial community structure in the birds from each lake, and the obvious differentiation in their gut microbial structures may indicate that the bacteria are highly sensitive to food sources at both lakes.

## Graphical abstract

We investigated the gut bacterial communities of lesser white‐fronted geese wintering at Shengjin Lake and Caizi Lake, China, the results showed that the differentiation significantly between the samples from both lakes. These findings suggest that diet may be an important driver of gut microbial community structure in the birds from each lake, and the obvious differentiation in their gut microbial structures may indicate that the bacteria are highly sensitive to food sources at both lakes.

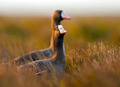

## 1 INTRODUCTION

Trillions of microbes inhabit the avian gut and form complex and diverse microbial communities (Wang et al., 2019). These communities make up a complex ecosystem of host and environmental factors, shaped by a series of dynamic and complex interactions between diet, lifestyle, and seasonal fluctuations (Dong, Xiang, Zhao, Song, & Zhou, 2019; Xiang, Zhang, Fu, Yan, & Zhou, 2019). Avian gut microbiota plays an important role in the host's physiology by contributing to functions such as host development, nutrient assimilation, vitamin synthesis, immune homeostasis, bile acid and sterol metabolism, and diseases in both nonhuman animals and humans alike (Ei, Dinan, & Cryan, 2014; Fukuda & Ohno, 2014; Kau, Ahern, Griffin, Goodman, & Gordon, 2011; McFall‐Ngai, Hadfield, Bosch, & Carey, 2013; O'Mahony, Clarke, Borre, & Dinan, 2015; Xiang et al., 2019; Zhao, Zhou, Dong, Cheng, & Song, 2017). Environment and diet are considered to be the main factors contributing to diversity in an animal's gut microbiota, and they strongly affect the gut microbiome composition. Gut microbial species and abundance tend to be more similar between closely related hosts than between more distantly related hosts in the same habitat (Eckburg et al., 2005; Yang, Deng, & Cao, 2016). However, gut microbiota varies among members of the same species living in different habitats and may also significantly differ among hosts of the same species from different geographical populations (Zhang et al., 2015).

Migratory bird species have complex diets, physiological traits, and life‐history strategies, and they provide an interesting opportunity for studying gut microbes (Dong et al., 2019; Kohl, 2012). Additionally, flying exerts a strong selective pressure on many aspects of their physiology, possibly changing the nature of their gut microbiota in the process (Yang et al., 2016). Avian gut microorganisms are important symbionts influencing the life of the host and, in turn, host birds may impact the gut microbial structure and function (Zhao et al., 2017). Thus, changes in the avian host diet as well as the environment greatly affect avian gut microbes (Dong et al., 2019; Kreisinger et al., 2017; Yang et al., 2016). However, knowledge about the avian gut microbiota lags behind that for other vertebrates, and previous studies on avian gut microbiota have mainly focused on economic and ornamental birds (Dewar et al., 2013; Wilkinson et al., 2017). Many studies that have focused on poultry have shown that diet and temporal stability can affect the gut microbiota of these birds (Kreisinger et al., 2017; Yang et al., 2016). However, few studies have reported on the gut microbiomes from wild birds, particularly those of long‐distance migratory waterbirds (Dong et al., 2019; Kohl, 2012; Pan & Yu, 2014; Waite & Taylor, 2014; Wang et al., 2016). Wild waterbirds remain less studied than other birds, and knowledge about gut microbiota in waterbirds of the same species from different geographic populations is scant, particularly for the gut microbiota from long‐distance migratory lesser white‐fronted geese (*Anser erythropus*) at their main wintering sites of Caizi and Shengjin Lakes along the middle and lower reaches of the Yangtze River in eastern China (Yang et al., 2016).

As long‐distance migratory waterbirds, lesser white‐fronted geese are an important wetland indicator species within the family Anatidae (order Anseriformes). The breeding area of this species extends from the Fennoscandian Lapland to northeastern Siberia and, in winter, to Japan, China and South Korea. In China, lesser white‐fronted geese primarily winter at Caizi and Shengjin Lakes along the middle and lower Yangtze River floodplains. Because of environmental heterogeneity, changes in lesser white‐fronted goose population dynamics may result from a reduction in suitable food resources and deterioration in the natural habitats at these two Chinese lakes (Zhang, Cao, Barter, & Fox, 2011; Zhao, Cao, Klaassen, Zhang, & Fox, 2015). Wintering lesser white‐fronted geese mainly inhabit lakes and associated wetlands. At Caizi Lake, the geese consume *Carex* meadow and subterranean tubers, whereas at Shengjin Lake, an extra dietary component, *Poaceae* spp., is present (Wang, Fox, Cong, Barter, & Cao, 2012; Zhao, Cong, Barter, Fox, & Cao, 2012). We believe that the different food sources consumed by wintering lesser white‐fronted geese affect their gut bacterial composition and structure; however, the distinctive gut bacteria belonging to this species have received little attention from researchers.

Caizi Lake (30.75°–30.97°N, 117.00°–117.15°E) and Shengjin Lake (30.25°–30.50°N, 116.92°–117.25°E) are shallow lakes in the middle and lower Yangtze River floodplains. Both lakes, which are designated internationally important wetlands, contain abundant aquatic resources and are important stopover and wintering grounds for many East Asian–Australasian migratory geese (Chen et al., 2011). We studied lesser white‐fronted geese from Caizi and Shengjin Lakes during their wintering period to compare their gut microbiota. High‐throughput sequencing of the 16S rRNA V3–V4 region and statistical analyses were performed to help describe the bacterial community structure and composition, and to determine whether the gut bacterial compositions exhibit the same patterns between the geese at the two wintering locations.

## 2 MATERIALS AND METHODS

### 2.1 Ethical standards

No animals were harmed during this research. All experimental procedures complied with current laws regarding animal welfare and research in China and were specifically approved by the Animal Research Ethics Committee of Anhui Medical University.

### 2.2 Sample collection

**FIGURE 1:**
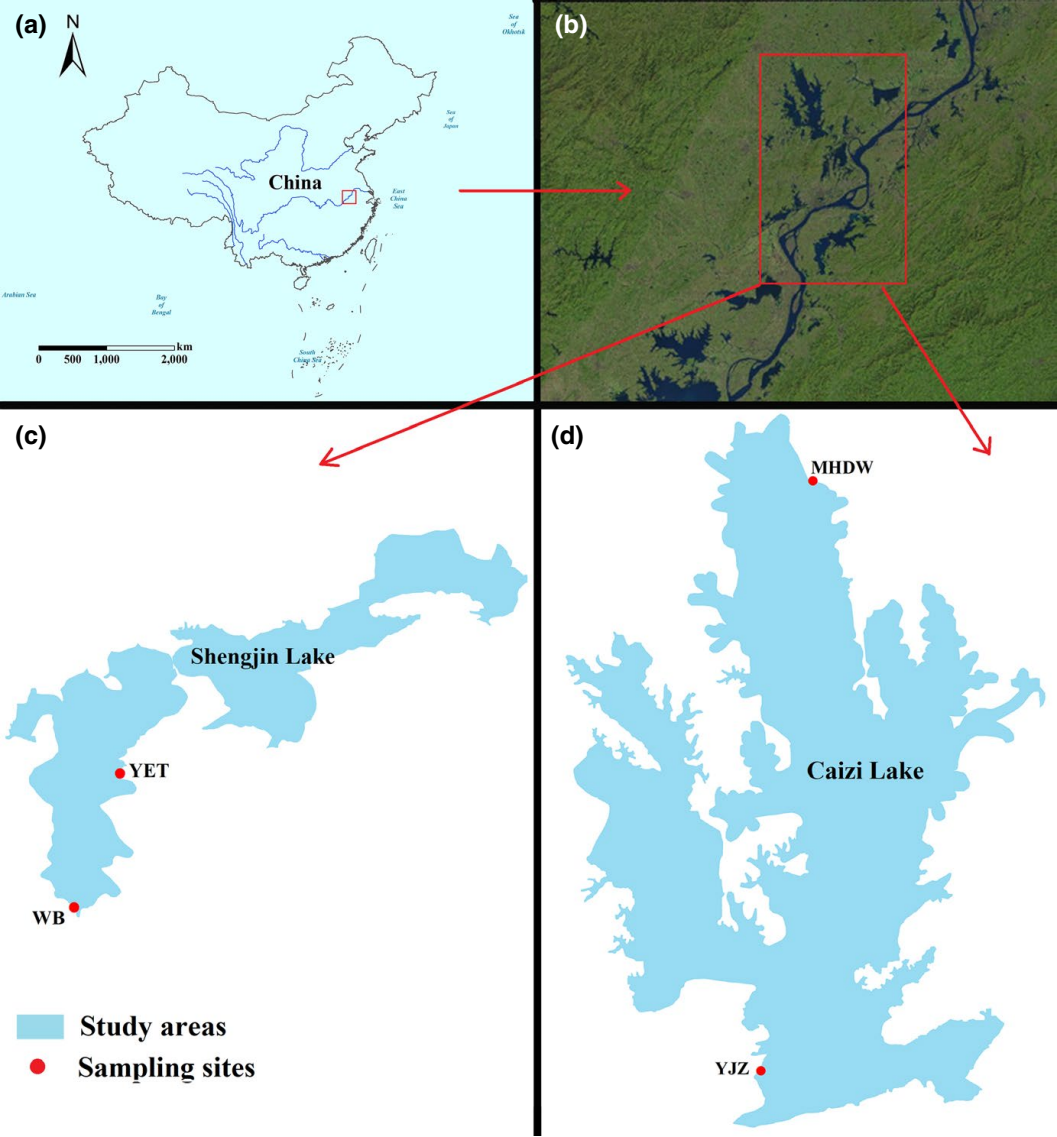
Fecal sampling sites for wintering lesser white‐fronted geese

Fecal samples were collected from Caizi Lake and Shengjin Lake, the two main wintering sites for lesser white‐fronted geese. Both lakes are river‐connected shallow lakes of the middle and lower Yangtze River (Figure 1). Both are globally important wintering habitats for migratory waterbirds on the East Asian–Australasian Flyway (Cao & Fox, 2009; Fox et al., 2011). Fecal samples were collected at foraging sites. Before the samples were collected, telescopes or binoculars were used to observe the geese and select large groups containing more than 150 birds. To avoid human disturbance and soil contamination, fresh fecal samples were collected immediately after the wild birds had finished foraging and had defecated. All samples were collected from the center of each fecal mass (Dong et al., 2019; Xiang et al., 2019), rapidly placed into sterile 50‐ml centrifuge tubes, transported to the laboratory, and stored at −80°C.

### 2.3 Fecal DNA extraction and avian species determination

DNA was extracted from the fecal samples using the Qiagen QIAamp R DNA Stool Mini Kit following the manufacturer's DNA isolation protocol. The extracted DNA was stored at −80°C. The cytochrome oxidase subunit 1 gene primer pair (BIRDF1: 5′‐TTC TCC AAC CAC AAA GAC ATT GGC AC‐3′ and BIRDR1: 5′‐ACG TGG GAG ATA ATT CCA AAT CCT G‐3′) was used for PCR amplification to determine the host species (Xiang et al., 2019). The cycling conditions were 95°C for 5 min, followed by 35 cycles of denaturation at 95°C for 30 s, annealing at 55°C for 45 s, extension at 72°C for 1.5 min, and a final extension at 72°C for 10 min. The PCR products were sequenced by Sangon Biotech Co. Ltd., and the resulting sequences were aligned in GenBank (https://www.ncbi.nlm.nih.gov/genbank/). All samples were confirmed to contain lesser white‐fronted goose DNA via sequencing analysis.

### 2.4 PCR amplification and Illumina MiSeq sequencing

The 338F/806R primer set, equipped with sequencing adapters and unique identifier tags, was used to amplify the bacterial 16S rRNA gene's V3–V4 regions from 30 fecal samples collected from lesser white‐fronted geese at Caizi and Shengjin Lakes (15 samples per lake). PCRs were conducted in 50 ml mixtures, each containing 200 mM deoxynucleoside triphosphates, 0.4 mM each of the forward and reverse primers, and 2 U of rTaq DNA polymerase (TaKaRa). The cycling conditions were 95°C for 5 min, followed by 30 cycles of 95°C for 30 s, 55°C for 45 s, and 72°C for 60 s, with a final extension at 72°C for 10 min. Tris‐boric acid‐ethylenediaminetetraacetic acid (2% w/v) agarose gels were used to assess the quality of the amplicons. Amplicons were purified using the MinElute PCR purification kit (Axygen), pooled at equal concentrations, and sequenced to identify the gut bacteria in them using the Illumina MiSeq platform at Oebiotech Co., Ltd. The raw data were submitted to the Sequence Read Archive at the NCBI database (https://www.ncbi.nlm.nih.gov/sra) under accession numbers SRR9641095–SRR9641124.

### 2.5 Data analysis

Raw sequencing data were prepared in FASTQ format. Trimmomatic software (version 0.35) was used to preprocess the paired‐end reads and detect and excise the ambiguous bases (N). Clean reads were subjected to primer sequence removal and clustered to generate operational taxonomic units (OTUs) using Vsearch software with a 97% similarity cutoff using USEARCH (version 7.1 http://drive5.com/uparse/). Differences in the bacterial community compositions between the two geese populations were analyzed via principal component analysis (PCA), and heatmaps using the vegan package (version 2.0‐2) in R v.2.8.1 were prepared. Rarefaction analysis and alpha‐diversity indices (abundance‐based coverage estimation [ACE], Chao1, Shannon and Simpson) were calculated using Mothur. Identification of the gut bacterial taxa that differed significantly between the two lakes was performed using linear discriminant analysis effect size (LEfSe), which uses the nonparametric Kruskal–Wallis rank sum test with the default setting (an alpha value of 0.05 and an effect size threshold of 2) to identify biomarkers. Functional predictions were made based on the 16S rRNA OTU membership using PICRUSt (Phylogenetic Investigation of Communities by Reconstruction of Unobserved States) according to the online protocol (http://picrust.github.io/picrust/).

## 3 RESULTS

### 3.1 General sequencing information

**FIGURE 2:**
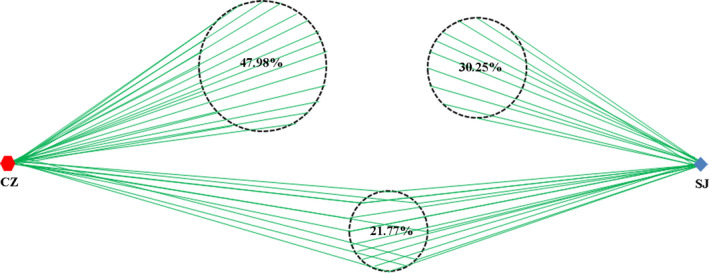
OTU richness for gut bacteria from lesser white‐fronted geese from Caizi (CZ) and Shengjin (SJ) Lakes

**FIGURE A1:**
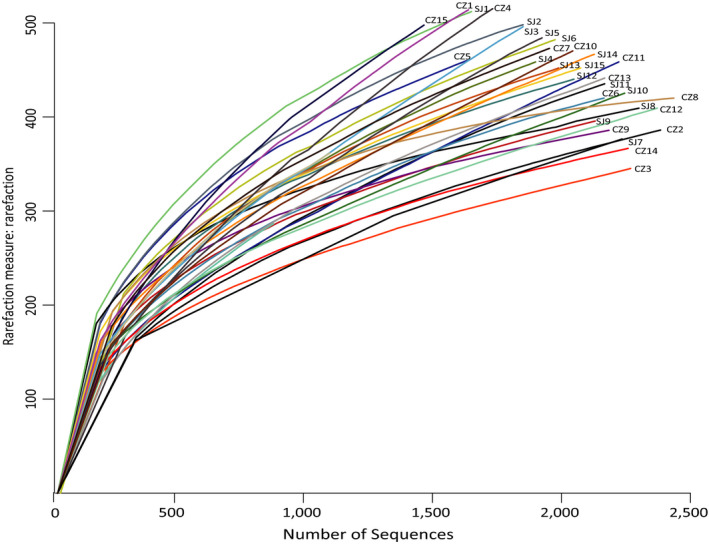
Rarefaction curves for 15 samples

Thirty fecal samples from lesser white‐fronted geese were collected from Caizi Lake and Shengjin Lake, and after processing, the 16S rRNA V3–V4 region gene was sequenced and analyzed. The Illumina MiSeq 2500 sequencing run produced 1,118,001 raw reads. Figure A1 shows the rarefaction curves for each sample. After removing low‐quality reads, 1,053,624 clean reads corresponding to 4,405 OTUs were retained. Each sample contained an average of 147 OTUs (range, 93–222 per sample) and 35,120 clean reads (range, 333,655–41,395 per sample). Of the OTUs, 21.77% were found in both populations. Geese from Caizi Lake had 47.98% unique OTUs, and geese from Shengjin Lake had 30.25% unique OTUs (Figure 2).

### 3.2 Gut bacterial alpha diversity

**FIGURE 3:**
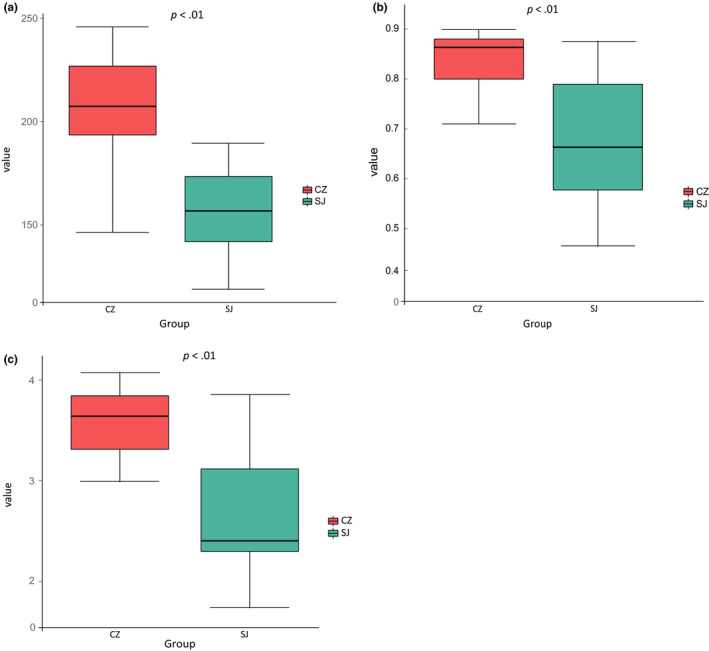
Alpha diversity of the gut bacteria from lesser white‐fronted geese at Caizi (CZ) and Shengjin (SJ) Lakes. Chao1 (a), Simpson (b), and Shannon (c) indices for the gut bacteria from the geese at each lake

**FIGURE 4:**
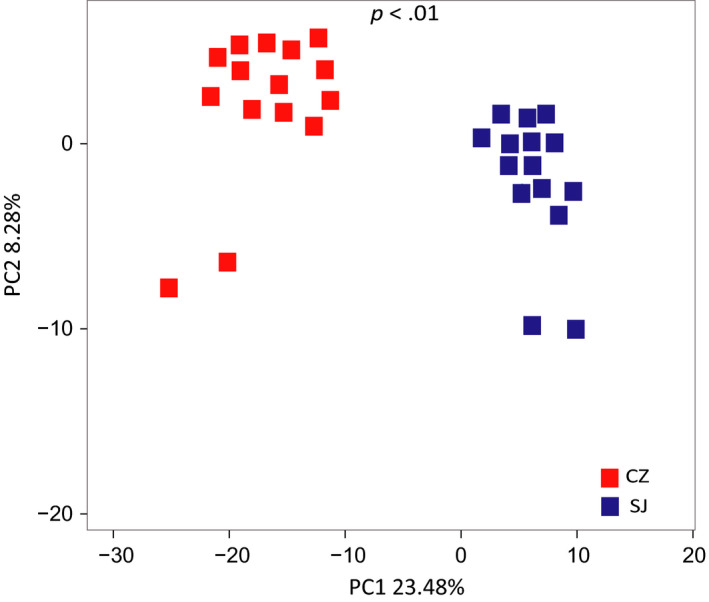
PCA of the weighted UniFrac distances for the species sampled from Caizi (CZ) and Shengjin (SJ) Lakes. Red: lesser white‐fronted geese from Caizi Lake; blue: lesser white‐fronted geese from Shengjin Lake

Gut bacterial α‐diversity was estimated via the observed Chao1, Simpson, and Shannon indices. The spatial distance and the Chao1, Simpson, and Shannon indices showed that the alpha diversity differed significantly between the Caizi Lake and Shengjin Lake samples (Figure 3). Alpha diversity for the Caizi Lake samples was significantly higher than for the Shengjin Lake samples (*p* < .01), as indicated by the number of observed OTUs (Figure 3). Furthermore, the fecal microbiota community compositions differed significantly between the guts of the lesser white‐fronted geese from both lakes. PCA analysis indicated that the samples were well matched with their lakes (Figure 4).

### 3.3 Gut bacterial community composition

**FIGURE 5:**
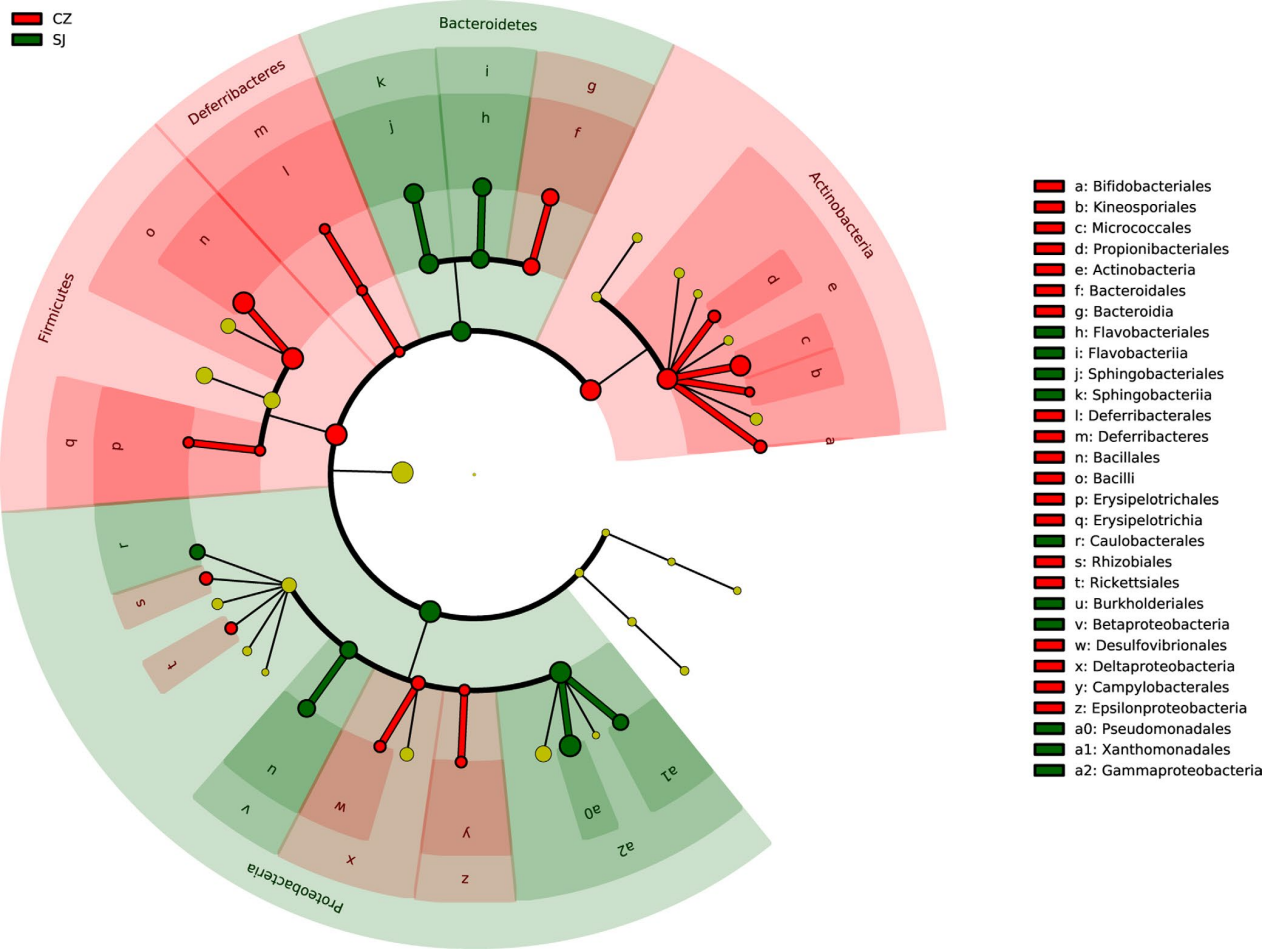
LEfSe analysis of the gut bacteria from lesser white‐fronted geese at Caizi (CZ) and Shengjin (SJ) Lakes (*p* < .05)

**FIGURE A2:**
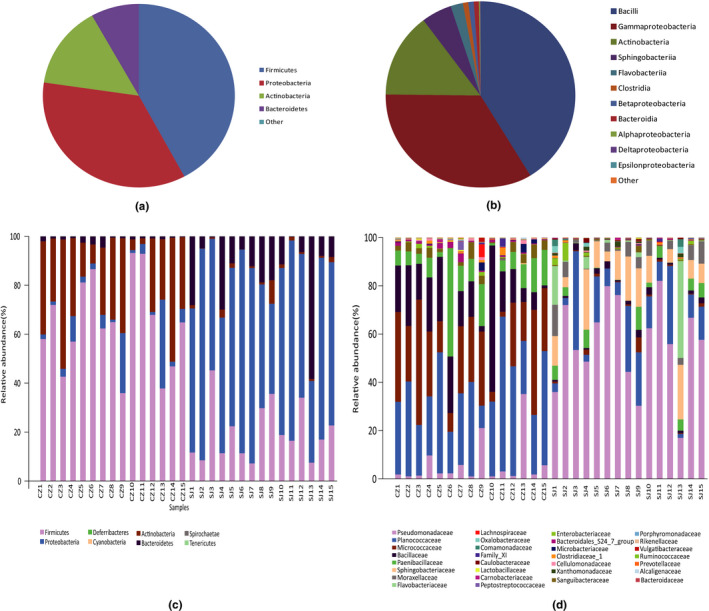
Relative abundances of gut bacteria at the phylum, class, and genus levels from lesser white‐fronted geese at Caizi and Shengjin Lakes. (a) Relative abundances of the dominant phyla. (b) Relative abundances of the dominant classes. (c) Relative abundances of the phyla. (d) Relative abundances of the classes for both lakes

The Caizi Lake samples contained eight phyla and 17 classes, whereas the Shengjin Lake samples contained seven phyla and 16 classes (Table 1). Firmicutes, Proteobacteria, Actinobacteria, and Bacteroidetes, the dominant gut bacterial phyla, accounted for 41.76%, 35.61%, 14.51%, and 8.12% of the OTUs, respectively. Deferribacteres, Cyanobacteria, Spirochaetae, and Tenericutes accounted for less than 0.001% of all bacteria, a result that was not statistically significant. The dominant class within the Firmicutes phylum was Bacilli (40.92%), Gammaproteobacteria (34.53%) was the dominant class within the Proteobacteria phylum, Actinobacteria (14.54%) was the dominant class within the Actinobacteria phylum, and Bacteroidia (0.85%) was the dominant class within the Bacteroidetes phylum (Figure A2). Our LEfSe analysis identified differences in the abundances of specific intestinal bacterial taxa in the guts of the lesser white‐fronted geese from the two lakes. Fibrobacteres and Actinobacteria phyla were significantly more abundant in the lesser white‐fronted geese from Caizi Lake (Figure 5), whereas Bacteroidetes and Proteobacteria were significantly more abundant in the geese from Shengjin Lake (Figure 5).

**TABLE 1:** Phyla and classes representing the gut bacteria from lesser white‐fronted geese at Caizi and Shengjin Lakes

Caizi Lake			Shengjin Lake		
Phyla	Classes		Phyla	Classes	
Firmicutes	Bacilli	Alphaproteobacteria	Firmicutes	Bacilli	Bacteroidia
Proteobacteria,	Gammaproteobacteria	Deltaproteobacteria	Proteobacteria	Gammaproteobacteria	Alphaproteobacteria
Actinobacteria,	Actinobacteria	Epsilonproteobacteria	Actinobacteria	Actinobacteria	Deltaproteobacteria
Bacteroidetes,	Sphingobacteriia	Erysipelotrichia	Bacteroidetes	Sphingobacteriia	Epsilonproteobacteria
Deferribacteres	Flavobacteriia	Deferribacteres	Deferribacteres	Flavobacteriia	Erysipelotrichia
Cyanobacteria,	Clostridia	Coriobacteriia	Cyanobacteria,	Clostridia	Deferribacteres
Spirochaetae	Betaproteobacteria	Chloroplast	Spirochaetae	Betaproteobacteria	Coriobacteriia
Tenericutes	Spirochaetes	Mollicutes		Spirochaetes	Chloroplast
	Bacteroidia				

**FIGURE 6:**
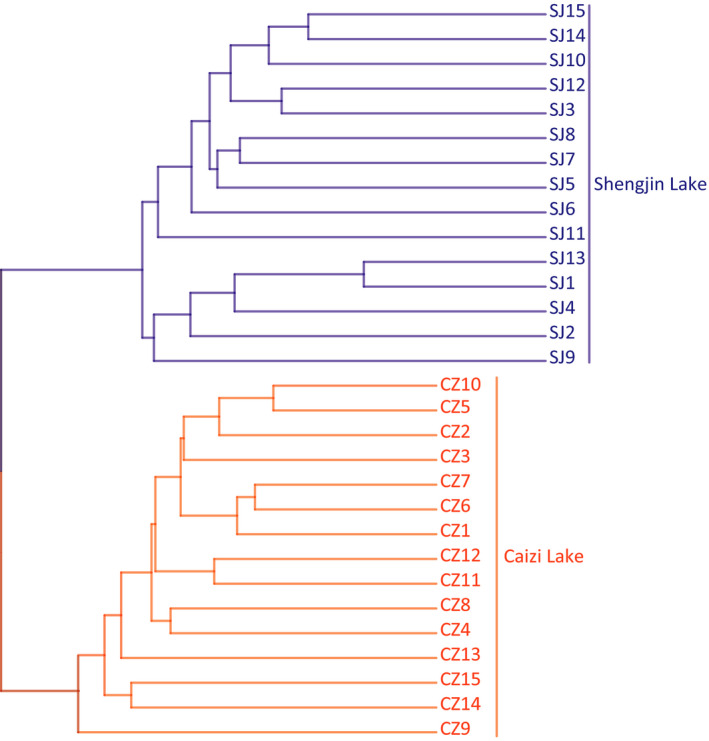
Phylogenetic relationships among the gut bacteria from 30 samples obtained from lesser white‐fronted geese at Shengjin and Caizi Lakes

**FIGURE A3:**
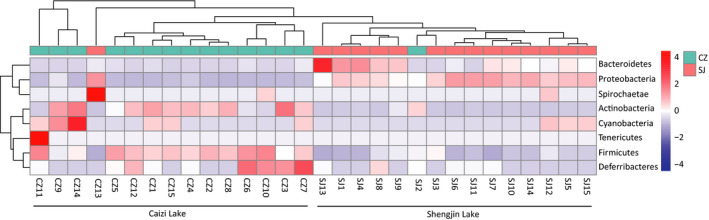
Heatmap for the gut bacterial samples from lesser white‐fronted geese at Caizi (CZ) and Shengjin (SJ) Lakes

The phylogenetic tree yielded two major branches: the first branch included the Shengjin Lake samples, and the second branch included the Caizi Lake samples (Figure 6). The heatmap analysis showed that all 30 samples were clustered into two major groups. Similar to the phylogenetic tree results, all 15 Caizi Lake samples were clustered together, and all 15 Shengjin Lake samples were clustered together (Figure A3).

### 3.4 PICRUSt analysis

**FIGURE 7:**
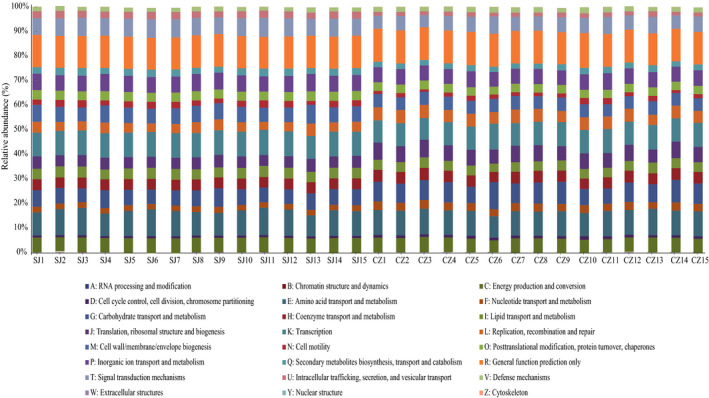
Functional predictions for all samples based on PICRUSt analysis

Overall, 24 KEGG (Kyoto Encyclopedia of Genes and Genomes) orthologs in the KEGG database were identified using PICRUSt (Phylogenetic Investigation of Communities by Reconstruction of Unobserved States) as a predictive exploratory tool for the gut bacterial communities from the lesser white‐fronted geese from Caizi Lake (Figure 7). Almost half of the major functions of the gut bacterial communities in the samples from the lesser white‐fronted geese from Caizi Lake were classified into multiple metabolism‐related groups (38.15%), including energy production and conversion (5.78%), amino acid transport and metabolism (10.05%), carbohydrate transport and metabolism (8.14%), coenzyme transport and metabolism (4.65%), lipid transport and metabolism (3.99%), inorganic ion transport and metabolism (6.19%), and secondary metabolite biosynthesis (2.38%). There were also 24 KEGG orthologs identified in the gut bacterial communities from the lesser white‐fronted geese from Shengjin Lake (Figure 7). Altogether, 42.01% of the major functions of the gut bacterial communities were classified into multiple metabolism groups for the Shengjin Lake samples, including energy production and conversion (5.97%), amino acid transport and metabolism (10.12%), carbohydrate transport and metabolism (6.42%), coenzyme transport and metabolism (4.40%), lipid transport and metabolism (4.31%), inorganic ion transport and metabolism (6.60%), and secondary metabolite biosynthesis (2.81%). The microbial functional classifications appear to be consistent in that most basic metabolic pathways were similar among the individual samples.

## 4 DISCUSSION

Lesser white‐fronted geese are obligate herbivores and long‐distance migratory waterbirds found in various ecosystems. However, the distinctive gut bacteria in these birds have received little attention (Yang et al., 2016). The present study is the first to explore the gut bacteria from lesser white‐fronted geese wintering at Caizi and Shengjin Lakes along the middle and lower reaches of the Yangtze River in eastern China. Our results suggest that a highly diverse gut bacterial community exists in the geese because the geese from the two sampling sites had different taxonomic and ecological bacterial compositions in their fecal samples, and diverse gut bacteria have been reported to display different adaptive mechanisms (Dong et al., 2019). In the present study, the gut bacterial composition and structure in lesser white‐fronted geese wintering at Shengjin and Caizi Lakes were explored. Differences in microbial community structures and interactions were identified. It seems likely that lesser white‐fronted geese may modify their digestion to adapt to variations in food availability between Caizi Lake and Shengjin Lake (Wu et al., 2018), and having a diverse gut microbiome may be one such adaptive mechanism.

Here, we found that only 21.77% of all the OTUs were common to geese at both lakes; thus, the community compositions and structures of the gut bacteria of the wintering lesser white‐fronted geese varied significantly in their microbial compositions between the two lakes. Firmicutes, Proteobacteria, and Actinobacteria dominated the gut bacterial compositions. All bacterial community assemblages showed significant phylogenetic clustering, indicating that the communities were strongly structured within their hosts (Dong et al., 2019; Xiang et al., 2019; Yang et al., 2016). The detailed compositions of these phyla clearly differed at the lower taxonomic level between the two groups. Among the Caizi Lake samples, the Firmicutes phylum mostly comprised Bacilli, while Proteobacteria mostly comprised the Gammaproteobacteria class. The class compositions of the Shengjin Lake samples differed from those of previous studies in other waterbirds, where it was revealed that the bacterial taxa showed strong host‐specific preferences, suggesting that host waterbirds play a crucial role in shaping the structure of their gut bacteria (Dong et al., 2019; Xiang et al., 2019). Our PICRUSt‐based functional predictions also showed that metabolic pathways accounted for the highest proportion of all the classified bacterial functions, a finding similar to that observed with the gut bacterial community from swan geese in Poyang Lake, China (Wu et al., 2018). Additionally, the bacterial community assemblages showed significant phylogenic clustering, indicating that the gut environment strongly influenced the bacterial community structure. These results suggest that environmental factors can influence the bacterial community composition of lesser white‐fronted geese and might be an important factor determining bacterial phylogenetic structuring.

The gut bacterial communities of wintering lesser white‐fronted geese may perform many important functions in their hosts. Supporting this assertion, the bacterial community assemblages from our geese showed significant alpha diversity, which indicates that the communities were strongly structured by gut environment filtering (Dong et al., 2019; Yang et al., 2016). The effect of environmental changes on the wintering lesser white‐fronted geese relating to bacterial alpha diversity was consistent between Shengjin and Caizi Lakes. Indeed, diet has been found to be an important driver of gut microbial community structure, and the obvious differentiation of the gut bacterial structures may indicate that the gut bacteria were highly sensitive to the food sources available at the two lakes (Yang et al., 2016). Caizi and Shengjin Lakes provide lesser white‐fronted geese with abundant and diverse food sources while wintering (Yang et al., 2016; Zhao et al., 2012). However, differences in the diets of the geese at the two lakes were large, with the lesser white‐fronted geese feeding almost exclusively on *Carex* spp. at Caizi Lake, whereas an extra *Poaceae* spp. component was identified at Shengjin Lake (Wang et al., 2012; Zhao et al., 2012). This suggests that food intake probably influenced the bacterial community compositions in the fecal samples from geese at two lakes and might be an important factor influencing the bacterial phylogenetic structure. The results from the PICRUSt and LEfSe analyses also provide support for diet as a factor influencing the compositions of the bacterial community in the fecal samples from geese at the two lakes. Lesser white‐fronted geese feed almost exclusively on *Carex* spp. at Caizi Lake, and Fibrobacteres and Actinobacteria were significantly more abundant in these geese. Carbohydrate is used as an energy source, and geese can digest simple carbohydrates and complex polysaccharides. Firmicutes and Actinobacteria, which are associated with high levels of carbohydrate metabolism, were also abundant in the Caizi Lake samples (Dong et al., 2019; Xiang et al., 2019). With respect to the Shengjin Lake geese, Bacteroidetes and Proteobacteria, which are associated with *Poaceae* spp. as food sources (Dong et al., 2019; Xiang et al., 2019), were significantly more abundant in the gut bacterial community from geese at this lake.

Fecal bacteria are often used as biomarkers for studying migratory connectivity in breeding and nonbreeding birds (Møller & Szép, 2011). Between Caizi Lake and Shengjin Lake, the geese in our study group consumed food that differed in type and quality. The environmental factors were comparatively homogeneous between the two lakes; however, the gut bacteria varied markedly between the geese from each lake. Furthermore, migratory birds also generally show strong‐site fidelity for both breeding and wintering locations, often returning to the same location each year during migration and in winter (Møller & Szép, 2011). This may be related to the fact that the lesser white‐fronted geese from Caizi Lake had migrated from a different breeding area than those from Shengjin Lake.

## CONFLICT OF INTERESTS

None declared.

## AUTHOR CONTRIBUTION

**Gang Liu:** Funding acquisition (supporting); Project administration (lead); Writing‐original draft (lead). **Zhizhong Gong:** Resources (lead); Software (lead). **Qingyue Li:** Data curation (lead); Investigation (lead).

## ETHICS STATEMENT

None required.

## Data Availability

All data are provided in full in the results section of this paper, all data was provided by NCBI database (https://www.ncbi.nlm.nih.gov/sra) under accession numbers SRR9641095–SRR9641124.
